# Supplementary material for: Reutilization of Silicon-Cutting Waste via Constructing Multilayer Si@SiO2@C Composites as Anode Materials for Li-Ion Batteries
Source: Nanomaterials (Basel). 2024 Apr 2;14(7):625. doi: 10.3390/nano14070625 (PMC11013368; doi:10.3390/nano14070625)
Supplement: Supplementary file 1 [file nanomaterials-14-00625-s001.zip › nanomaterials-2936256-supplementary.pdf]

Supplementary Materials

Reutilization of Silicon-Cutting Waste via Constructing Multilayer Si@SiO$_2$@C Composites as Anode Materials for Li-Ion Batteries

Yi Sun, Jingyi Wu, Xingjie Chen and Chunyan Lai *

Shanghai Key Laboratory of Materials Protection and Advanced Materials in Electric Power,
Shanghai University of Electric Power, Shanghai 200090, China;
15797926318@163.com (Y.S.); wujingyi0728@163.com (J.W.);
a1317358861@163.com (X.C.)
* Correspondence: laichunyan@shiep.edu.cn

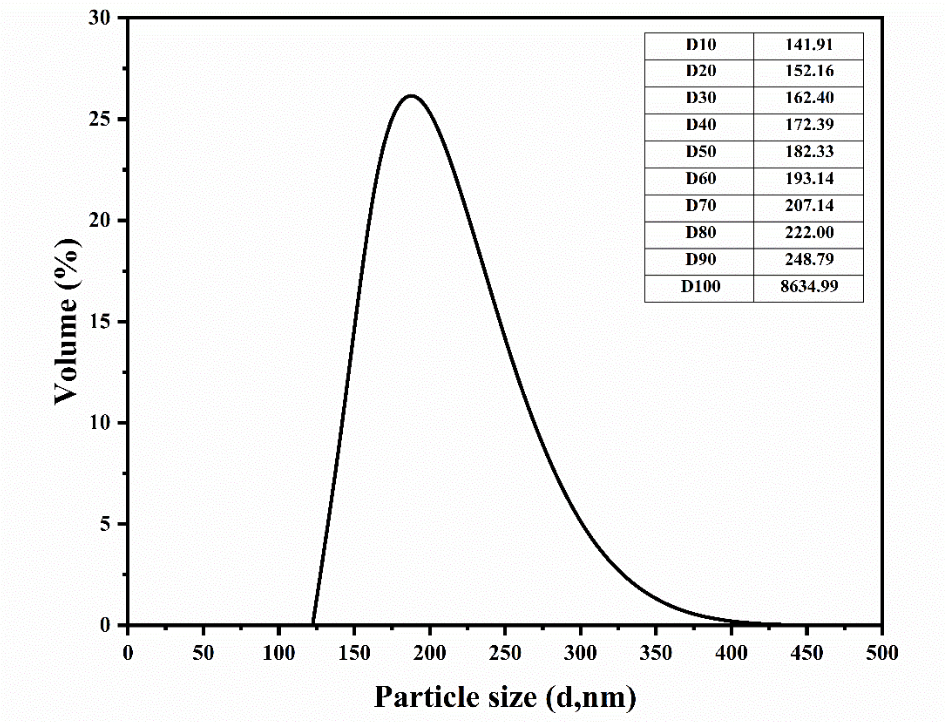

Figure S1. Particle size distribution of SCW.

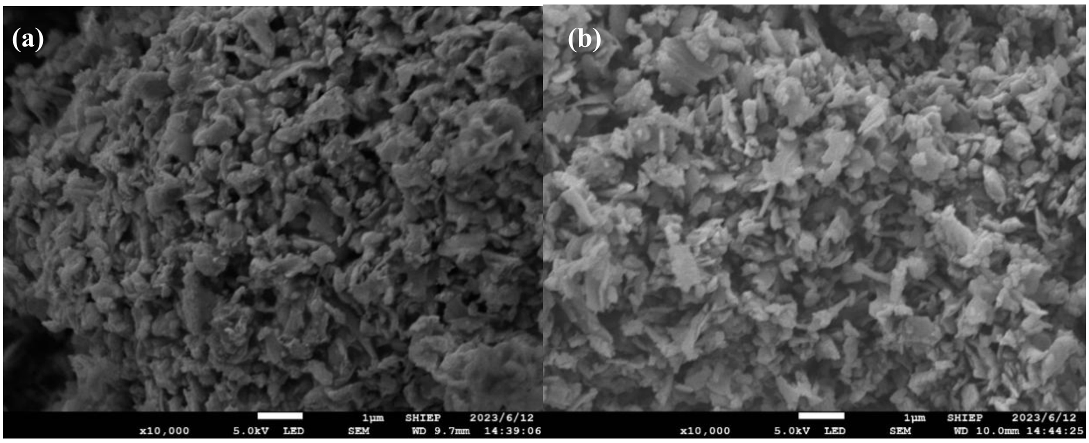

Figure S2. SEM images of (a) Si@SiO_2@C-2 and (b) Si@SiO_2@C-4.

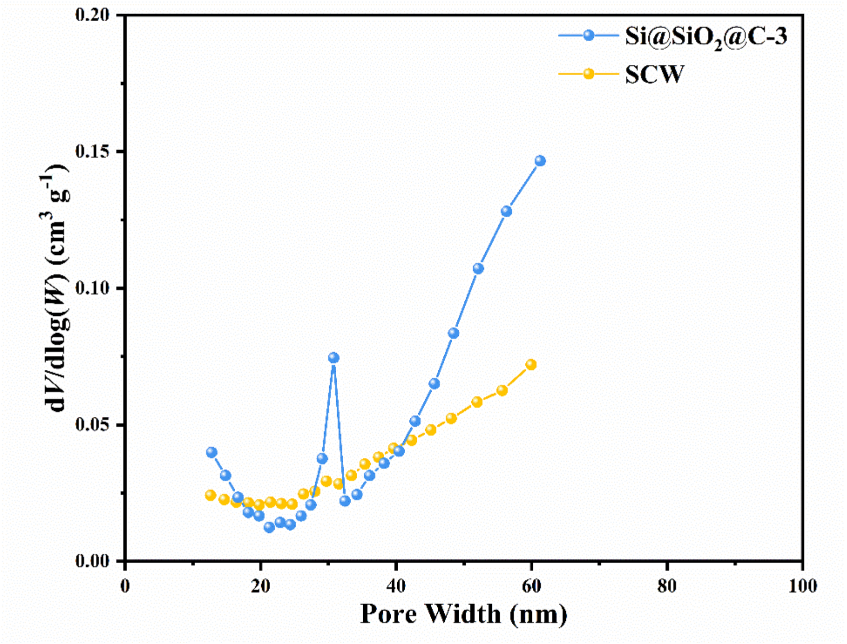

Figure S3. Pore size distribution of SCW and Si@SiO_2@C-3.

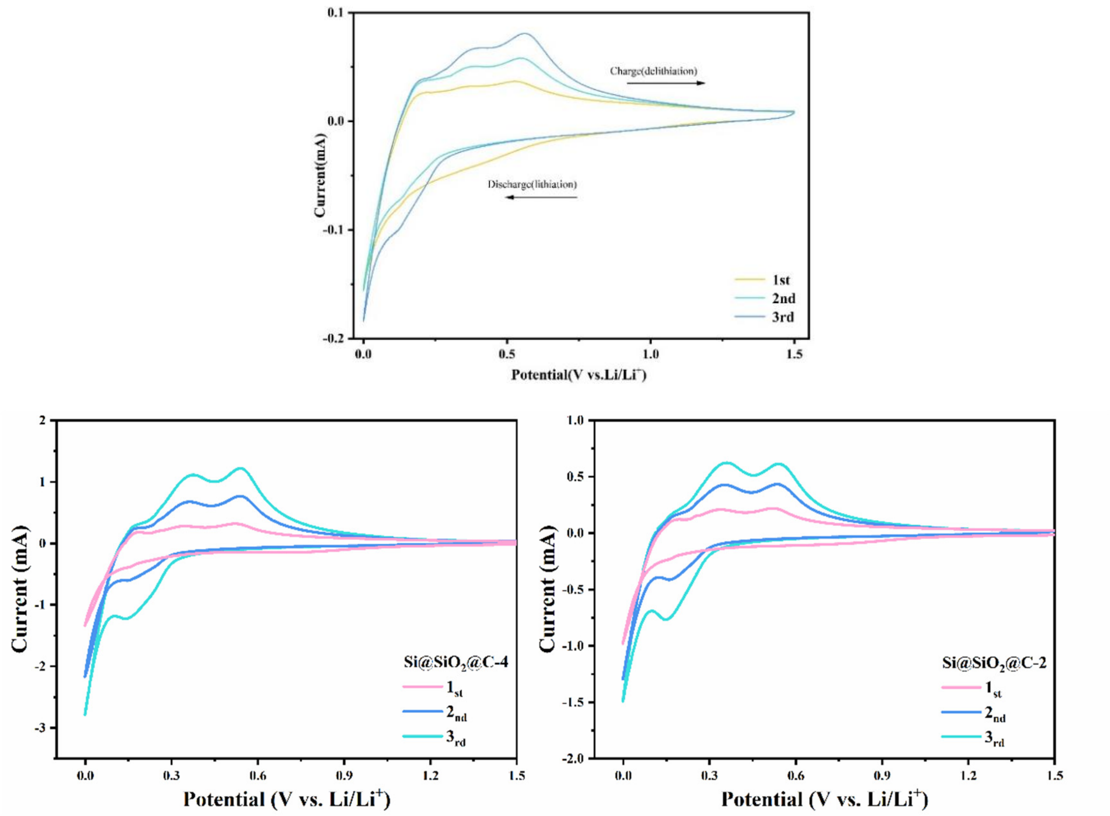

Figure S4. The initial three CV curves of SCW, $Si@SiO_2@C$-4 and $Si@SiO_2@C$-2 at 0.1 mV s^{-1}.

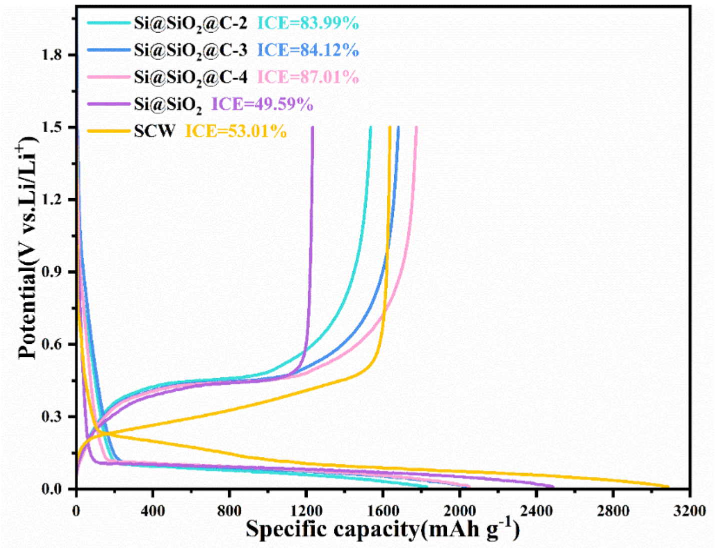

Figure S5. The initial GCD curves of SCW, Si@SiO$_2$, Si@SiO$_2$@C-4, Si@SiO$_2$@C-3 and Si@SiO$_2$@C-2 at 0.1 mV s^{-1}.

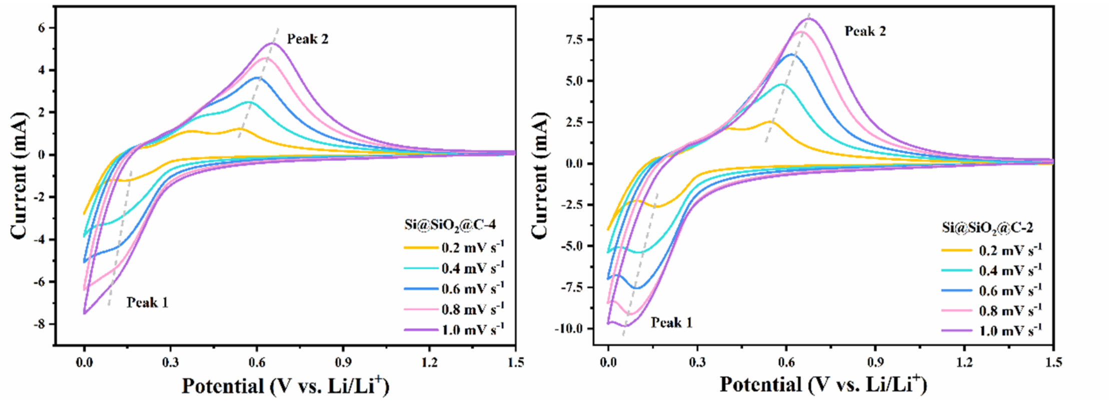

Figure S6. The CV curves of Si@SiO$_2$@C at 0.2, 0.4, 0.6, 0.8 and 1.0 mV s^{-1} scan rates.

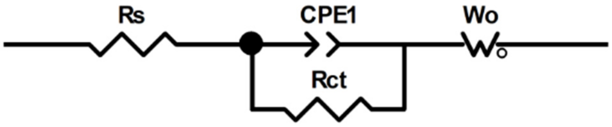

Figure S7. Equivalent circuit model linked with EIS curves.

Table S1. XRF results of SCW.

Sample EA	**Si**	**C**	**O**	**Ni**	**Cl**	**Fe**	**Cu**	**Ca**	**Al**
**Weight (%)**	82.4808	9.1417	8.1182	0.0679	0.0622	0.0513	0.0313	0.0273	0.0194

Table S2. BET specific surface area, pore volume, and BJH pore size of SCW and Si@SiO_2@C-3.

Sample	S_{BET} (m^2 g^{-1})	V_{total} (cm^3 g^{-1})	Average pore size (BJH) (nm)
**SCW**	114.49	0.26	8.31
**Si@SiO_2@C-3**	60.56	0.13	9.29

Table S3. The R_{ct} and Warburg values of SCW, $Si@SiO_2@C$-2, $Si@SiO_2@C$-3 and $Si@SiO_2@C$-4.

Sample	R_{ct} (ohm)	σ ($\Omega\ S^{-1/2}$)
**SCW**	37.17	236.16
**$Si@SiO_2@C$-2**	26.56	55.39
**$Si@SiO_2@C$-3**	18.55	32.11
**$Si@SiO_2@C$-4**	57.19	69.26
